# Assessment of change and persistence of youth psychosocial status reported by youth and their guardians during the COVID-19 pandemic: A MyHEARTSMAP study

**DOI:** 10.1371/journal.pone.0329898

**Published:** 2025-08-08

**Authors:** Melissa L. Woodward, Morgan W. Wolsey, Sophia Shalchy-Tabrizi, Jeffrey N. Bone, Tyler Black, Quynh Doan

**Affiliations:** 1 Department of Pediatrics, University of British Columbia, Vancouver, British Columbia, Canada; 2 British Columbia Children’s Hospital Research Institute, Vancouver, British Columbia, Canada; 3 Department of Psychiatry, University of British Columbia, Vancouver, British Columbia, Canada; NYU Grossman School of Medicine: New York University School of Medicine, UNITED STATES OF AMERICA

## Abstract

**Background:**

The pediatric mental health crisis pre-dated the COVID 19 pandemic with rates of mental health visits to pediatric emergency departments steadily increasing for the last decade. The COVID-19 pandemic has profoundly impacted children and adolescents and understanding the trajectory of their psychosocial status is important for appropriate resource allocation and policy planning.

**Methods:**

MyHEARTSMAP is a digital self-assessment mental health evaluation that examines four major psychosocial domains: psychiatry, social, function, and youth health. Children and adolescents throughout British Columbia, and their guardians, completed the baseline assessment between August 2020 and July 2021 (51.8% completed by guardian only, 40.2% youth and guardians, 7.9% youth only). Both children and their guardians repeated the MyHEARTSMAP evaluation three-months after their baseline. Patient demographics and psychosocial concerns were statistically described and compared between baseline and follow-up. A logistic regression model assessed the influence of baseline scores and demographic factors on follow-up severity.

**Results:**

241 of 424 participants (56.8%) completed both the baseline and three-month follow-up. The majority of participants reported no change overtime across the psychosocial domains. Both improvement and decline occurred in each domain, with a greater proportion of psychosocial states improving rather than worsening, for all domains. Higher severity of psychosocial concerns reported at baseline indicated a greater likelihood of psychosocial concerns at 3-month follow-up for psychiatric, social and function concerns. Demographic, pandemic, and support service variables were not associated with psychosocial trajectories.

**Conclusions:**

The severity of youth mental health concerns in British Columbia remained consistent through three-month follow up, despite the changing nature of the COVID-19 pandemic during this period. Greater persistence of psychosocial concerns with increased severity highlights the need for early intervention to prevent worsening mental health. Community support is needed for youth experiencing mental health concerns to address mild psychosocial concerns before presentation at the emergency department.

## Introduction

The prolonged challenges of the COVID-19 pandemic brought significant disruption to the lives of children and adolescents. High rates of depression, anxiety and other psychiatric concerns among youth have been observed during the pandemic [1–3]. Children who were older, girls, or LGBTQ+ demonstrated greater risk of these mental health concerns and worry about COVID-19, academic stress, conflict with parents, and prior mental health concerns were key risk factors for youth [4–7].

Concern over the long-term impacts of the pandemic on the mental health of children has shone a spotlight on the declining state of youth mental health and insufficient resources prior to the COVID-19 pandemic [8]. Mental health visits to the BC Children’s Hospital pediatric emergency department increased 85.8% from 2002 to 2012, compared to a 27.5% increase in non-mental health visits, with similar trends seen in other institutions [9,10]. Rates of involuntary hospitalization for mental health disorders increased from 2008 to 2018 with adolescent girls being among the most at risk [11]. More recent data indicate that pediatric mental health emergency presentations declined or stayed the same early in the pandemic, with later increases, particularly for girls [12–14].

During the pandemic, using the MyHEARTSMAP digital self-assessment tool, the researchers reported high prevalence of mental health concerns in the community, beyond those presenting to emergency departments [4]. This study aims to evaluate the persistence of these community mental health concerns among children and adolescents over three months during the COVID-19 pandemic and the potential impact of accessing recommended community mental health support services and resources. Understanding the trajectory of mental health concerns and factors that may contribute to changing mental health is important for policy and resource planning to ensure adequate support for youth experiencing mental health concerns.

## Methods

### Study recruitment

Children and youth aged 6–17 years and their guardians living in British Columbia (BC) were enrolled between August 10^th^, 2020 and June 25^th^, 2021. Participants were recruited virtually through partnerships with youth- and family-oriented organizations, digital health and education networks, and identity groups, throughout BC. Online media-driven recruitment was complemented with traditional media outlets such as newspaper, radio, television, and physical bulletins to reach families with limited or no internet access. Voluntary response sampling was used for initial recruitment, with purposive sampling through a private recruitment company to recruit a geographically diverse sample representative of the BC population. Details of the recruitment procedure have been published [15]. Participants gave informed electronic consent documented through a secure online survey while on a phone call with a trained research assistant who notified a research clinician (pediatric emergency nurse) of the presence of any psychosocial concerns. The research clinician would contact the family to assess the situation and guide them to access appropriate care or contact the appropriate authorities in the case of imminent threats to safety. Youth participants provided assent while their parents or guardians gave consent for their participation. Participants who were unable to communicate in English were excluded. Approval was granted by the University of British Columbia Children’s and Women’s Health Centre Research Ethics Board.

### MyHEARTSMAP assessment

The MyHEARTSMAP psychosocial self-assessment tool is a digital health evaluation for youth, completed by the youth or their guardian. Adapted from HEARTSMAP, a clinical tool for the assessment and management of mental health-related presentations in emergency departments, MyHEARTSMAP has been validated for self-assessment for universal screening use in community [16–18]. Psychosocial measures are assessed for severity using a 4-point Likert-type scale from 0 (no concern) to 3 (severe concern) across 10 sections: home, education and activities, alcohol and drugs, relationships and bullying, thoughts and anxiety, safety, sexual health, mood, abuse, and current professional resources. These sections are grouped into four domains based on resources recommendation: psychiatric, social, function, and youth health, with summative severity scores ranging from 0 (none), 1–3 (mild), 4–6 (moderate), and 7 and above (severe). Domain-specific resource recommendations are triggered by any non-zero score within the sections and participants are asked about any established support services and resources for that concern. Multiple recommendations per domain could be triggered. These recommendations can include referral to online resources, mental health teams, redirection to a general practitioner, protective agencies and services, and youth health services. Further details have been published previously [15].

### Study design

The study followed a sequential-cohort design with cross-sectional components. At baseline, consented participants filled out an online survey to collect demographic- and pandemic-related information, along with the self-administered MyHEARTSMAP tool to complete their psychosocial assessment. Study materials could be filled out by a guardian, child, or both at the discretion of the participants. Entries that reported severe or acute safety issues triggered alerts to an on-call pediatric emergency research nurse and directed participants to crisis lines and emergency departments for urgent help. The research nurse, trained in mental health assessments, contacted such participants or their guardians directly to ensure that they followed through with the recommendations to access emergency services, as per standard clinical care. Reminders to complete the survey were sent through email or phone calls. At three-months, participants completed the MyHEARTSMAP tool again, and a follow-up questionnaire that collected information regarding their pandemic experience and support service access.

### Objectives & measures

Our primary objective is to report the change in frequency of self- and/or guardian-identified psychosocial issues over a three-month period for children and adolescents in BC during the COVID-19 pandemic. Our secondary objective is to determine associations between change in severity of psychosocial concerns and participant characteristics (demographic and pre-determined pandemic-related variables, including access to support services). Baseline findings and an assessment of support service access have been published previously [4,19]. To increase sensitivity, the higher score was used when both guardian and youth severity scores were available. Average annual income quartile was derived from the Canada Revenue Agency Individual Tax Statistics, using the first 3 digits of participants’ residential postal codes. The impact of accessing support services recommended by the MyHEARTSMAP tool-embedded algorithm was evaluated, generated based on the severity and pattern of scores within each domain and participant resource access, on psychosocial trajectory for each domain.

### Statistical analysis

Descriptive statistics were used to summarise demographic and outcome measures of participants who completed the study at the three-month mark. The demographic distribution for those who did not complete the study were compared to those who did to assess for possible selection bias (S1 Table). The change in frequency of psychosocial issues was expressed as the proportion of participants who experienced an improvement, no change, or worsening of symptoms compared to baseline. An ordinal logistic regression model was generated to assess the influence of baseline scores on follow-up severity. The adjusted model controlled for age, gender, ethnicity and income level. Demographic characteristics, pandemic-related experiences (ex. death of close relative due to COVID-19), and support service variables (ex. ability to access recommended support service) were included in the model that may have been relevant to the change in psychosocial severity to assess their impact. Model results are summarised as odds ratios and 95% confidence intervals. All analyses were conducted an expert biostatistician (JNB) using R statistical software version 4.0.3 [20]. R is a free, open-source software and programming language capable of performing any statistical model, data analysis, and data visualization.

## Results

### Participants

**Fig 1:**
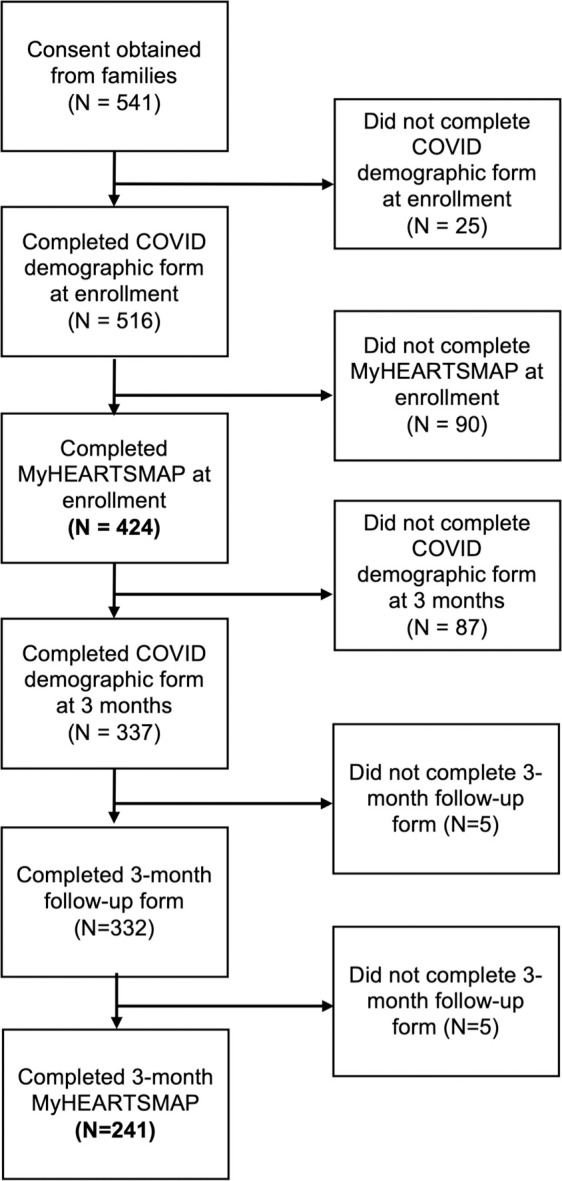
Flowchart of study data collection.

A total of 241 participants completed the MyHEARTSMAP 3-month follow-up survey, 56.8% of the 424 baseline MyHEARTSMAP participants (Fig 1). With similar rates to the baseline assessments, 97 (40.2%) were completed by both the guardian and child, 125 (51.8%) by guardian only, and 19 (7.9%) by youth only. The demographics of these participants can be found in Table 1. The mean age of the youth was 10.7 years (SD 3.26). Participants were 51.0% (123/241) female, 48.5% (117/241) male, and 0.41% (1/241) preferred not to specify. The recruited participants represented all provincial health authorities: 35.3% (85/241) from Fraser Health, 29.9% (72/241) from Vancouver Coastal Health, 10.0% (24/241) from Interior Health, 17.4% (42/241) from Island Health and, 7.5% (18/241) from Northern Health, similar to the population proportion across these regions. A comparison of the distribution of health authority representation and a comparison of demographic variables between 3-month respondents and non-respondents is available in S1 Table. No significant differences were observed between participant demographic characteristics between those who did and did not complete the 3-month follow-up assessment.

**Table 1:** Participant demographics at 3-month follow-up.

CHARACTERISTIC	N = 241
**Age:** mean years (SD)	10.7 (3.3)
**Sex** N (%)	
Female	123 (51.0)
Male	117 (48.5)
Prefer not to say	1 (0.41)
**Gender** N (%)	
Girl/Young woman	117 (48.5)
Boy/Young man	119 (49.4)
Other	5 (2.07)
**Ethnicity** N (%)	
Asian	18 (7.47)
Black or African	1 (0.41)
Indigenous	6 (2.49)
Hispanic	1 (0.41)
Middle Eastern	1 (0.41)
Multiethnic	37 (15.4)
White	177 (73.4)
**Health Authority** N (%)	
Fraser	85 (35.3)
Interior	24 (9.96)
Island	42 (17.4)
Northern	18 (7.47)
Vancouver Coastal	72 (29.9)
**Current type of school attendance** N (%)	
At home (full time)	34 (14.1)
In person (full time)	118 (49.0)
Part time (in person)	34 (14.1)
No school or formal education program	27 (11.2)
Summer holiday	28 (11.6)
**Guardian employment status** N (%)	
Employed work at home	36 (14.9)
Employed work outside of home	110 (45.6)
Self-employed work at home	37 (15.4)
Self-employed work outside of home	10 (4.15)
Unemployed	48 (19.9)
**Neighbourhood income** median CAD (SD)	51852 (11315)

CAD = Canadian dollar; NA = not applicable; SD = standard deviation.

### Primary and secondary outcomes

**Fig 2:**
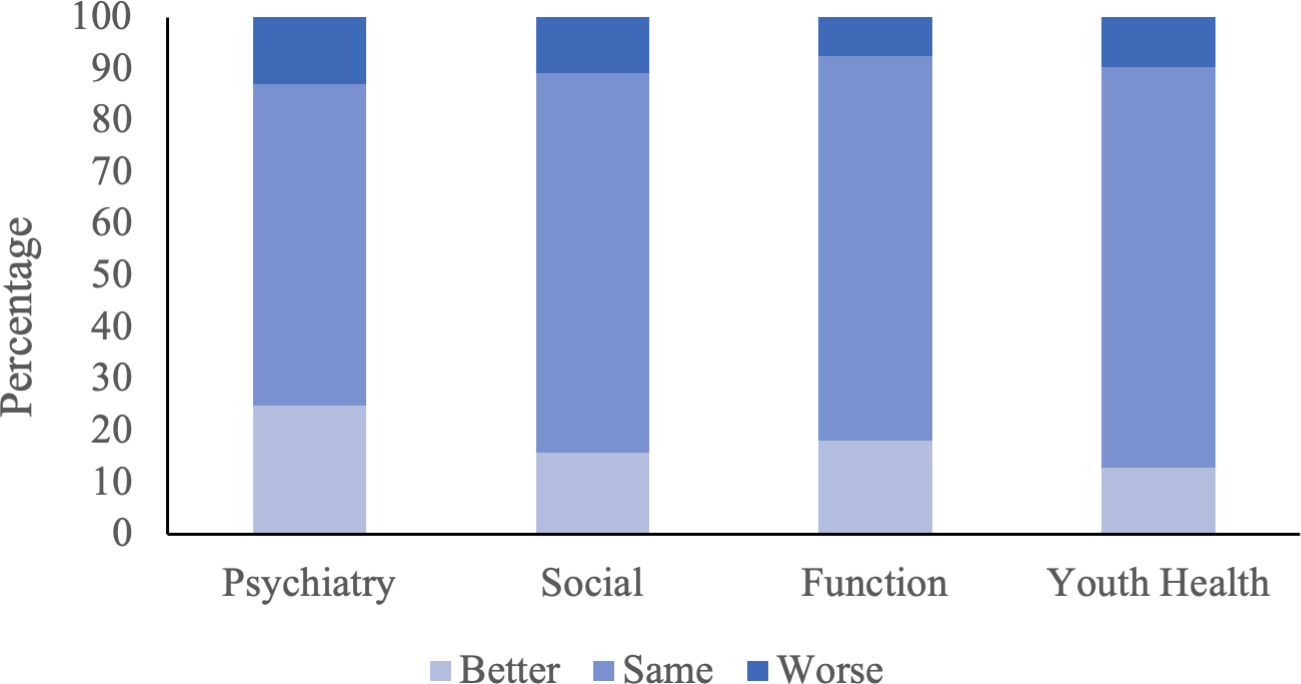
Change in psychosocial domain between baseline and 3-month follow-up.

The distribution of severity scores across the psychosocial domains at baseline and 3-month follow-up is listed in Table 2. The majority of youth endorse some psychosocial concerns at 3-month follow-up for psychiatric (mild – 61.0%, moderate – 12.0%, severe – 2.5%) and social (mild – 66.0%, moderate – 1.7%) domains with a smaller proportion of concerns reported for the function (mild – 45.6%, moderate – 4.2%), and youth health (mild – 38.6%, moderate – 0.4%) domains. Assessing individual trajectories, the proportion of youth who experienced greater or reduced severity of their psychosocial concerns across the four domains between the baseline assessment and 3-month follow-up was summarized (Fig 2 and S2 Table). Within the psychiatry domain, 24.9% of youth improved at least one level of severity, 62.2% remained the same, and 12.9% became more severe. In the social domain, 15.8% of participants improved, 73.4% remained the same, and 10.8% experienced deterioration. In the function domain, 18.3% of participants saw an improvement, 74.3% remained the same, and 7.5% worsened. In the youth health domain, 12.9% of participants improved, 77.6% remained the same, and 9.5% worsened. S3 Table provides a summary of changes for each domain and score with their calculated proportions per severity.

**Table 2:** Distribution of severity scores for psychosocial domains at baseline and 3-month follow-up (N = 241).

Psychosocial Domain	Severity Score	Baseline N (%)	3-Month Follow-Up N (%)
Psychiatry	None	38 (15.8%)	59 (24.5%)
Psychiatry	Mild	156 (64.7%)	147 (61.0%)
Psychiatry	Moderate	42 (17.4%)	29 (12.0%)
Psychiatry	Severe	5 (2.1%)	6 (2.5%)
Social	None	73 (30.3%)	78 (32.4%)
Social	Mild	157 (65.2%)	159 (66.0%)
Social	Moderate	11 (4.6%)	4 (1.7%)
Function	None	96 (39.8%)	121 (50.2%)
Function	Mild	132 (54.8%)	110 (45.6%)
Function	Moderate	13 (5.4%)	10 (4.15%)
Youth Health	None	139 (57.7%)	147 (61.0%)
Youth Health	Mild	100 (41.5%)	93 (38.6%)
Youth Health	Moderate	2 (0.8%)	1 (0.4%)

The likelihood of an individual reporting the presence of psychosocial concerns for each domain (a severity above “none”) given their baseline severity score is reported in Table 3. An individual who reported mildly severe psychiatric concerns at baseline had 3.9 times the odds of reporting psychiatric concerns of any severity at 3-month follow-up compared to someone without psychiatric concerns at baseline (OR = 3.9, 95% CI = 1.9, 8.5). Those who reported a moderate psychiatric score at baseline had 38 times the odds (OR = 38.0, 95% CI = 13.1, 114.6) of reporting psychiatric concerns at follow-up, and someone who reported severe had 35.3 the odds (OR = 35.3, 95% CI = 4.2, 277.4) of reporting persistent psychiatric concerns. Individuals with mild social concerns at baseline had 8.3 times the odds (OR = 8.3, 95% CI = 4.3, 16.6) of reporting social concerns at follow-up and moderate social concerns at baseline had 172.0 times the odds (OR = 172.0, 95% CI = 21.7, 1694.6) of reporting persistent social concerns. Individuals with mild function concerns at baseline had 15.6 times the odds (OR = 15.6, 95% CI = 7.8, 33.4) of experiencing function concerns at three-month follow-up compared to individuals without function concerns at baseline and individuals with moderate function concerns at baseline had 214.8 times the odds (OR = 214.8, 95% CI = 46.8, 1078.6) of experiencing persistent function concerns. Individuals with mild youth health domain concerns had 14.5 times the odds (OR = 14.5, 95% CI = 7.5, 29.9) of experiencing youth health concerns at follow-up, while an individual who reported moderate youth health concerns at baseline had 4.1 times the odds (OR = 4.1, 95% CI = 0.2, 105.5) of reporting youth health concerns at three-month follow-up. The unadjusted odds ratios are available in S4 Table. Demographic characteristics, pandemic experiences, and support service access were not significantly associated with the trajectory of psychosocial concerns from baseline to three-month follow-up or the likelihood of persistent concerns in the logistic regression model.

**Table 3:** Adjusted odds ratios of individuals who experience baseline psychosocial concerns at each severity of experiencing psychosocial concerns within that same domain at three-month follow-up.

Psychosocial Domain	Baseline Severity	OR (95% CI)	p-value
Psychiatry	Mild	3.9 (1.9, 8.5)	<0.001
Psychiatry	Moderate	38.0 (13.1, 114.6)	<0.001
Psychiatry	Severe	35.3 (4.2, 277.4)	<0.001
Social	Mild	8.3 (4.3, 16.6)	<0.001
Social	Moderate	172.0 (21.7, 1694.6)	<0.001
Function	Mild	15.6 (7.8, 33.4)	<0.001
Function	Moderate	214.8 (46.8, 1078.6)	<0.001
Youth Health	Mild	14.5 (7.5, 29.9)	<0.001
Youth Health	Moderate	4.1 (0.2, 105.5)	0.329

CI = confidence interval; OR = odds ratio.

Adjusted for age, gender, ethnicity and income level.

## Discussion

Generally, psychosocial concerns of children and youth observed at baseline persisted at three-month follow-up across all psychosocial domains. The majority of participants reported psychiatric and social concerns while nearly half reported function concerns. This consistency in psychosocial concerns despite changes in COVID-19-related health guidelines and personalised recommendations for community mental health support services indicate that these concerns are not isolated to early periods of the pandemic and more care is needed in addressing the current youth mental health crisis [4,8].

This persistence in psychosocial concerns followed a dose-dependent relationship such that individuals with greater severity of concerns were more likely to endorse psychosocial concerns at three-month follow-up. This relationship was true for the psychiatric, social and function domains, but not the youth health domain likely due to the very small number of participants who experienced moderate youth health concerns at both baseline and three-month follow-up. This highlights the importance of early intervention for youth mental health concerns, even if only mildly severe, as these concerns are relatively consistent and may worsen, becoming more likely to persist. Notably, where change in psychosocial concerns did occur, a greater proportion of individuals experienced improvement compared to worsening across all four psychosocial domains. Protective factors including maintaining social connections, as well as benefits of the MyHEARTSMAP assessment itself including fostering communication between parents and guardians and their children, and seeking out recommended support services, may have contributed to this improvement [19,21].

### Limitations

The ability to follow this sample of children and youth longitudinally lends credence to the consistency of these psychosocial concerns in community and the importance of addressing these concerns prior to presentation at the emergency department, but there are some limitations. Due to the reduced sample size at follow-up, the researchers were unable to determine any significant impact of demographic characteristics, pandemic experiences, and support service variable on the change or persistence of psychosocial social concerns. Further research would be valuable as to highlight youth who may be at particular risk for worsening mental health concerns in need of targeted intervention. It is important to highlight that due to a lack of pre-pandemic scoring, this study is not able to assess changes brought about by the pandemic itself. Changes within the three-month period may not be solely due to the COVID-19 pandemic and the consistency of youth mental health concerns prior to 2020 suggests that there are many factors that contribute to youth psychosocial concerns that pre-date the pandemic. The focus of this analysis is instead on mental health changes during the pandemic and the potential impact of the MyHEARTSMAP assessment and the individualized community mental health service recommendations. Three-month follow-up was assessed to evaluate the ability to access recommended community mental health services in a timely manner. Longer follow-up and sequential cohort studies will be important for understanding the overall trajectory of youth mental health as they age into adulthood and as younger children enter their school years. Further detail into the types of community mental health supports that most benefit children and possibilities for addressing barriers around time, availability, and parental education will also be highly beneficial for policy planning and resource allocation.

## Conclusions

The majority of youth continue to experience psychiatric and social concerns three-months following their baseline MyHEARTSMAP assessment. Most individuals had no change in the severity of their psychosocial concerns with some experiencing improvement and a smaller proportion experiencing worsening over the three-month period. Individuals with greater severity psychiatric, social, and function concerns were more like to experience persistent concerns. The consistency of community youth mental health concerns highlights the need to address the current youth mental health crisis and provide appropriate resources for children and their guardians.

## Supporting information

S1 Table Comparison of baseline participant demographic characteristics for those who completed 3-month follow-up and those who did not. (DOCX)

S2 Table Change in psychosocial domain between baseline and 3-month follow-up. (N = 241). (DOCX)

S3 Table Proportion of individuals at each severity score for both baseline and 3-month follow-up across the psychosocial domains. (N = 241). (DOCX)

S4 Table Unadjusted odds ratios of individuals who experience baseline psychosocial concerns at each severity of experiencing psychosocial concerns within that same domain at three-month follow-up. (DOCX)

## References

[1] Jones EAK, Mitra AK, Bhuiyan AR. Impact of COVID-19 on Mental Health in Adolescents: A Systematic Review. Int J Environ Res Public Health. 2021;18(5):2470. doi: 10.3390/ijerph18052470 33802278 PMC7967607

[2] Nearchou F, Flinn C, Niland R, Subramaniam SS, Hennessy E. Exploring the Impact of COVID-19 on Mental Health Outcomes in Children and Adolescents: A Systematic Review. Int J Environ Res Public Health. 2020;17(22):8479. doi: 10.3390/ijerph17228479 33207689 PMC7698263

[3] Meherali S, Punjani N, Louie-Poon S, Abdul Rahim K, Das JK, Salam RA, et al. Mental Health of Children and Adolescents Amidst COVID-19 and Past Pandemics: A Rapid Systematic Review. Int J Environ Res Public Health. 2021;18(7):3432. doi: 10.3390/ijerph18073432 33810225 PMC8038056

[4] Woodward ML, Hossain A, Chun A, Liu C, Kilyk K, Bone JN, et al. Evaluating the psychosocial status of BC children and youth during the COVID-19 pandemic: A MyHEARTSMAP cross-sectional study. PLoS One. 2023;18(3):e0281083. doi: 10.1371/journal.pone.0281083 37000793 PMC10065280

[5] Magson NR, Freeman JYA, Rapee RM, Richardson CE, Oar EL, Fardouly J. Risk and Protective Factors for Prospective Changes in Adolescent Mental Health during the COVID-19 Pandemic. J Youth Adolesc. 2021;50(1):44–57. doi: 10.1007/s10964-020-01332-9 33108542 PMC7590912

[6] Hertz MF, Barrios LC. Adolescent mental health, COVID-19, and the value of school-community partnerships. Injury Prevention. 2021;27(1):85–6. doi: 10.1136/injuryprev-2020-044050 33172840

[7] Panchal U, Salazar de Pablo G, Franco M, Moreno C, Parellada M, Arango C, et al. The impact of COVID-19 lockdown on child and adolescent mental health: systematic review. Eur Child Adolesc Psychiatry. 2023;32(7):1151–77. doi: 10.1007/s00787-021-01856-w 34406494 PMC8371430

[8] Barican J, Lou Y, Schwartz C, Zheng Y, Georgiades K, Waddell C. Prevalence of childhood mental disorders in high-income countries: a systematic review and meta-analysis to inform policymaking. Evid Based Ment Health. 2022;25(1):36–44. doi: 10.1136/ebmental-2021-300277 34281985 PMC8788041

[9] Mapelli E, Black T, Doan Q. Trends in Pediatric Emergency Department Utilization for Mental Health-Related Visits. J Pediatr. 2015;167(4):905–10. doi: 10.1016/j.jpeds.2015.07.004 26256019

[10] Sheridan DC, Spiro DM, Fu R, Johnson KP, Sheridan JS, Oue AA, et al. Mental Health Utilization in a Pediatric Emergency Department. Pediatr Emerg Care. 2015;31(8):555–9. doi: 10.1097/PEC.0000000000000343 25834957 PMC4526317

[11] Loyal JP, Lavergne MR, Shirmaleki M, Fischer B, Kaoser R, Makolewksi J, et al. Trends in Involuntary Psychiatric Hospitalization in British Columbia: Descriptive Analysis of Population-Based Linked Administrative Data from 2008 to 2018. Can J Psychiatry. 2023;68(4):257–68. doi: 10.1177/07067437221128477 36200433 PMC10037746

[12] Poonai N, Freedman SB, Newton AS, Sawyer S, Gaucher N, Ali S, et al. Emergency department visits and hospital admissions for suicidal ideation, self-poisoning and self-harm among adolescents in Canada during the COVID-19 pandemic. CMAJ. 2023;195(36):E1221–30. doi: 10.1503/cmaj.220507 37722746 PMC10506508

[13] Hernández-Calle D, Andreo-Jover J, Curto-Ramos J, García Martínez D, Valor LV, Juárez G, et al. Pediatric mental health emergency visits during the COVID-19 pandemic. Scand J Child Adolesc Psychiatr Psychol. 2022;10(1):53–7. doi: 10.2478/sjcapp-2022-0005 35836474 PMC9238432

[14] Saunders NR, Toulany A, Deb B, Strauss R, Vigod SN, Guttmann A, et al. Acute mental health service use following onset of the COVID-19 pandemic in Ontario, Canada: a trend analysis. CMAJ Open. 2021;9(4):E988–97. doi: 10.9778/cmajo.20210100 34785528 PMC8598241

[15] Virk P, Chun A, Liu Q, Doan Q. Recruiting and engaging youth and families in mental health research: lessons learned during the COVID-19 pandemic. 2022. https://methods.sagepub.com/case/recruiting-engaging-youth-families-mental-health-research-lessons-covid-19

[16] Li BCM, Wright B, Black T, Newton AS, Doan Q. Utility of MyHEARTSMAP in youth presenting to the emergency department with mental health concerns. Journal of Pediatrics. 2021;235:124–9. doi: 10.1016/j.jpeds.2021.03.062 33819465

[17] Virk P, Stenstrom R, Doan Q. Reliability testing of the HEARTSMAP psychosocial assessment tool for multidisciplinary use and in diverse emergency settings. Paediatr Child Health. 2018;23(8):503–8. doi: 10.1093/pch/pxy017 30842695 PMC6242031

[18] Virk P, Laskin S, Gokiert R, Richardson C, Newton M, Stenstrom R, et al. MyHEARTSMAP: development and evaluation of a psychosocial self-assessment tool, for and by youth. BMJ Paediatr Open. 2019;3(1):e000493. doi: 10.1136/bmjpo-2019-000493 31414065 PMC6668754

[19] Shalchy-Tabrizi S, Woodward ML, Virk P, Wolsey MW, Kang M, Bone JN, et al. Evaluating access to child and youth mental health resources following psychosocial screening during the COVID-19 pandemic. Submitted manuscript. 2023.

[20] R Core Team. R: A Language and Environment for Statistical Computing. Vienna, Austria: R Foundation for Statistical Computing. 2023.

[21] Hertz MF, Kilmer G, Verlenden J, Liddon N, Rasberry CN, Barrios LC, et al. Adolescent Mental Health, Connectedness, and Mode of School Instruction During COVID-19. The Journal of Adolescent Health. 2022;70(1):57–63. doi: 10.1016/j.jadohealth.2021.10.021 34930571 PMC8531003

