# Supplementary material for: Assessment of change and persistence of youth psychosocial status reported by youth and their guardians during the COVID-19 pandemic: A MyHEARTSMAP study
Source: PLoS One. 2025 Aug 8;20(8):e0329898. doi: 10.1371/journal.pone.0329898 (PMC12334015; doi:10.1371/journal.pone.0329898)
Supplement: S1 Table — (DOCX) [file pone.0329898.s001.docx]

**S1 Table. Comparison of baseline participant demographic characteristics for those who completed 3-month follow-up and those who did not.**

|  | **Complete** | **Incomplete** | **p-value** |
| --- | --- | --- | --- |
| **CHARACTERISTIC** | **N = 241** | **N = 183** |  |
| **Age:** mean years (SD) | 10.7 (3.3) | 11.1 (3.4) | 0.186 |
| **Sex** N (%) |  |  | 0.912 |
| Female | 123 (51.0) | 96 (52.5) |  |
| Male | 117 (48.5) | 87 (47.5) |  |
| Prefer not to say | 1 (0.4) | 0 (0.0) |  |
| **Gender** N (%) |  |  | 0.919 |
| Girl/Young woman | 117 (48.5) | 93 (50.8) |  |
| Boy/Young man | 119 (49.4) | 86 (47.0) |  |
| Other | 5 (2.1) | 4 (2.2) |  |
| **Ethnicity** N (%) |  |  | 0.650 |
| Asian | 18 (7.5) | 12 (6.6) |  |
| Black or African | 1 (0.4) | 3 (1.6) |  |
| Indigenous | 6 (2.5) | 6 (3.3) |  |
| Hispanic | 1 (0.4) | 2 (1.1) |  |
| Middle Eastern | 1 (0.4) | 1 (0.6) |  |
| Multiethnic | 37 (15.4) | 35 (19.1) |  |
| White | 177 (73.4) | 124 (67.8) |  |
| **Health Authority** N (%) |  |  | 0.910 |
| Fraser | 85 (35.3) | 64 (35.0) |  |
| Interior | 24 (10.0) | 17 (9.3) |  |
| Island | 42 (17.4) | 40 (22.0) |  |
| Northern | 18 (7.5) | 13 (7.1) |  |
| Vancouver Coastal | 72 (29.9) | 49 (26.9) |  |
| **Guardian employment status** N (%) |  |  | 0.051 |
| Employed work at home | 36 (14.9) | 33 (18.0) |  |
| Employed work outside of home | 110 (45.6) | 97 (53.0) |  |
| Self-employed work at home | 37 (15.4) | 13 (7.1) |  |
| Self-employed work outside of home | 10 (4.2) | 11 (6.0) |  |
| Unemployed | 48 (19.9) | 29 (15.8) |  |
| **Current type of school attendance** N (%) |  |  |  |
| At home (full time) | 34 (14.9) | 31 (17.0) |  |
| In person (full time) | 118 (49.0) | 83 (45.4) |  |
| Part time (in person) | 34 (14.1) | 30 (16.4) |  |
| No school or formal education program | 27 (11.2) | 28 (15.3) |  |
| Summer holiday | 28 (11.6) | 11 (6.01) |  |
| **Neighbourhood income** median CAD (SD) | 51852 (11315) | 53832 (14847) | 0.281 |

CAD = Canadian dollar; NA = not applicable; SD = standard deviation
