# Supplementary material for: Assessment of change and persistence of youth psychosocial status reported by youth and their guardians during the COVID-19 pandemic: A MyHEARTSMAP study
Source: PLoS One. 2025 Aug 8;20(8):e0329898. doi: 10.1371/journal.pone.0329898 (PMC12334015; doi:10.1371/journal.pone.0329898)
Supplement: S2 Table — (DOCX) [file pone.0329898.s002.docx]

**S2 Table. Change in psychosocial domain between baseline and 3-month follow-up. (N = 241)**

| **Psychosocial Domain** | **Change** | **Percent (N)** |
| --- | --- | --- |
| Psychiatry | Better | 24.9% (60) |
| Psychiatry | Same | 62.2% (150) |
| Psychiatry | Worse | 12.9% (31) |
| Social | Better | 15.8% (38) |
| Social | Same | 73.4% (177) |
| Social | Worse | 10.8% (26) |
| Function | Better | 18.3% (44) |
| Function | Same | 74.3% (179) |
| Function | Worse | 7.5% (18) |
| Youth Health | Better | 12.9% (31) |
| Youth Health | Same | 77.6% (187) |
| Youth Health | Worse | 9.5% (23) |
