# Supplementary material for: Assessment of change and persistence of youth psychosocial status reported by youth and their guardians during the COVID-19 pandemic: A MyHEARTSMAP study
Source: PLoS One. 2025 Aug 8;20(8):e0329898. doi: 10.1371/journal.pone.0329898 (PMC12334015; doi:10.1371/journal.pone.0329898)
Supplement: S3 Table — (DOCX) [file pone.0329898.s003.docx]

**S3 Table. Proportion of individuals at each severity score for both baseline and 3-month follow-up across the psychosocial domains. (N = 241)**

| Psychosocial Domain | Baseline Score | 3-Month Follow-Up Score | N | Proportion* |
| --- | --- | --- | --- | --- |
| Psychiatry | None | None | 22 | 0.58 |
| Psychiatry | None | Mild | 15 | 0.39 |
| Psychiatry | None | Moderate | 1 | 0.03 |
| Psychiatry | Mild | None | 35 | 0.22 |
| Psychiatry | Mild | Mild | 110 | 0.71 |
| Psychiatry | Mild | Moderate | 10 | 0.06 |
| Psychiatry | Mild | Severe | 1 | 0.01 |
| Psychiatry | Moderate | None | 2 | 0.05 |
| Psychiatry | Moderate | Mild | 19 | 0.45 |
| Psychiatry | Moderate | Moderate | 17 | 0.4 |
| Psychiatry | Moderate | Severe | 4 | 0.1 |
| Psychiatry | Severe | Mild | 3 | 0.6 |
| Psychiatry | Severe | Moderate | 1 | 0.2 |
| Psychiatry | Severe | Severe | 1 | 0.2 |
| Social | None | None | 49 | 0.67 |
| Social | None | Mild | 24 | 0.33 |
| Social | Mild | None | 29 | 0.18 |
| Social | Mild | Mild | 126 | 0.8 |
| Social | Mild | Moderate | 2 | 0.01 |
| Social | Moderate | Mild | 9 | 0.82 |
| Social | Moderate | Moderate | 2 | 0.18 |
| Function | None | None | 82 | 0.85 |
| Function | None | Mild | 14 | 0.15 |
| Function | Mild | None | 37 | 0.28 |
| Function | Mild | Mild | 91 | 0.69 |
| Function | Mild | Moderate | 4 | 0.03 |
| Function | Moderate | None | 2 | 0.15 |
| Function | Moderate | Mild | 5 | 0.38 |
| Function | Moderate | Moderate | 6 | 0.46 |
| Youth Health | None | None | 117 | 0.84 |
| Youth Health | None | Mild | 22 | 0.16 |
| Youth Health | Mild | None | 29 | 0.29 |
| Youth Health | Mild | Mild | 70 | 0.7 |
| Youth Health | Mild | Moderate | 1 | 0.01 |
| Youth Health | Moderate | None | 1 | 0.5 |
| Youth Health | Moderate | Mild | 1 | 0.5 |

*Proportion of baseline respondents for that domain at that severity level at baseline for each severity level at 3-month follow-up.
