# Supplementary material for: Assessment of change and persistence of youth psychosocial status reported by youth and their guardians during the COVID-19 pandemic: A MyHEARTSMAP study
Source: PLoS One. 2025 Aug 8;20(8):e0329898. doi: 10.1371/journal.pone.0329898 (PMC12334015; doi:10.1371/journal.pone.0329898)
Supplement: S4 Table — (DOCX) [file pone.0329898.s004.docx]

**S4 Table. Unadjusted odds ratios of individuals who experience baseline psychosocial concerns at each severity of experiencing psychosocial concerns within that same domain at three-month follow-up.**

| **Psychosocial Domain** | **Baseline Severity** | **OR (95% CI)** | **p-value** |
| --- | --- | --- | --- |
| Psychiatry | Mild | 4.5 (2.2, 9.6) | <0.001 |
| Psychiatry | Moderate | 51.6 (18.8, 147.8) | <0.001 |
| Psychiatry | Severe | 49.1 (6.6, 352.2) | <0.001 |
| Social | Mild | 9.1 (4.9, 17.4) | <0.001 |
| Social | Moderate | 186.0 (25.0, 1728.7) | <0.001 |
| Function | Mild | 14.5 (7.6, 29.6) | <0.001 |
| Function | Moderate | 211.3 (46.9, 1010.2) | <0.001 |
| Youth Health | Mild | 13.1 (7.1, 25.0) | <0.001 |
| Youth Health | Moderate | 5.3 (0.2, 127.2) | 0.243 |

CI= confidence interval; OR = odds ratio
